# Socioeconomic inequality in different phenotypes of childhood obesity and its determinants in Iran: a Blinder-Oaxaca decomposition method

**DOI:** 10.1186/s12889-022-13997-x

**Published:** 2022-08-20

**Authors:** Zohreh Mahmoodi, Paramjit Gill, Mostafa Qorbani, Nami Mohammadian Khonsari, Ali Sheidaei, Ramin Heshmat, Motahar Heidari-Beni, Roya Kelishadi

**Affiliations:** 1 Warwick Medical School, University of Warwick, Coventry, UK; 2 Social Determinants of Health Research Center, Alborz University of Medical Sciences, Karaj, Iran; 3 Non-Communicable Diseases Research Center, Alborz University of Medical Sciences, Karaj, Iran; 4 Chronic Diseases Research Center, Endocrinology and Metabolism Population Sciences Institute, Tehran University of Medical Sciences, Tehran, Iran; 5 Department of Epidemiology and Biostatistics, School of Public Health, Tehran University of Medical Sciences, Tehran, Iran; 6 Department of Pediatrics, Child Growth and Development Research Center, Research Institute for Primordial Prevention of Non-Communicable Disease, Isfahan University of Medical Sciences, Isfahan, Iran

**Keywords:** Inequality, Adolescents, Oaxaca-Blinder decomposition, Iran, Obesity

## Abstract

**Background:**

Childhood obesity has become a significant public health issue worldwide. Socioeconomic status is among its key determinants. This study examined the socioeconomic inequality in different phenotypes of childhood obesity at the national level in Iran.

**Methods:**

This national, multistage school cross-sectional study was undertaken in 2015 on 14,400 students aged 7–18 years from urban and rural areas of 30 provinces of Iran. Using principal component analysis, socioeconomic status (SES) was categorized into tertiles. SES inequality in different phenotypes of obesity (i.e., generalized obesity", "abdominal obesity", and combined obesity) was estimated using the concentration index. The determinants of this inequality were assessed by the Blinder-Oaxaca decomposition method.

**Results:**

Overall, 14,274 students completed the study (response rate: 99%). The mean age was 12.28 years, 50.6% were boys, and 71.42% lived in urban areas. The prevalence of generalized obesity and abdominal obesity was 20.8% and 11.3%, respectively. The concentration index for different phenotypes of obesity was positive, indicating that inequality is more common amongst the low SES groups. High SES, being male, living in a rural, and having a positive family history of obesity were associated with general obesity. Moderate physical activity and living in a rural area were associated with abdominal obesity. In addition, living in a rural area, having a high SES, being male, and having a positive family history of obesity were associated with combined obesity.

**Conclusion:**

According to the present study findings, all childhood obesity phenotypes were more prevalent in Iranian children with high SES. Therefore, due to obesity and other diseases, it is essential to implement environmental changes in addition to designing macro-educational programs and prevention strategies.

## Background

The increasing prevalence of childhood obesity (generalized and abdominal) has become a health concern worldwide in recent years. The rapidly rising obesity prevalence in children can lead to adult morbidity and mortality. Nonetheless, its prevalence has doubled in many countries since the 1980s [1].

Childhood obesity prevalence varies by country; for example, in Iran, 18%, Jordan 4%-8%, America, 11%-19% of children and adolescents aged 5–19 years, and in Spain, 37%-39% of children aged 6–11 years are overweight or obese [2–4].

The increasing risk of various associated co-morbidities with obesity, such as adulthood obesity, diabetes, and non-communicable diseases, has been confirmed in previous studies, implementing the importance of its prevention and control [2–5].

Nevertheless, managing childhood obesity remains a challenge and may relate to targeting adipose tissue. Thus, body mass index (BMI) could help assess the amount of fat [5]. However, individuals with obesity have heterogeneous phenotypes, each associated with various health conditions. Metabolically unhealthy obesity (MUO) defines subjects at high risk of metabolic diseases, and metabolically healthy obesity (MHO) refers to individuals with obesitythat are at a low risk of developing cardiometabolic disorders. Compared with MUO individuals, MHOs have more abdominal fat but less visceral fat mass and fat collection in their liver and skeletal muscles [6]. Additionally other types of obesity such as normal weight obesity (NWO) have been described. In this type of obesity, regardless of having a normal BMI, due to a high fat percentage, the individual is considered as obese; and is at increased risk of obesity related morbidity and mortality; however, since their BMI falls within the normal range, they may go undetected until obesity related morbidities appear [7]. Hence a thorough understanding of childhood obesity, BMI and their determinants is needed to fight this pandemic.

Some determinants affecting obesity are known as “social determinants”, which are a diverse range of social, economic and environmental factors that impact children's health. Therefore, to prevent childhood obesity, assessing their social determinants of health is essential [2, 8]; and although the associations between socioeconomic status (SES) and some growth disorders have been documented among the adult population, studies on children are limited and inconsistent. Therefore, recognizing these factors that impact and cause inequality regarding this subject is very important [8].

Inequality is one of the realities understood in people's lives as it affects people's lifestyles. This concept is generally defined according to individuals' different needs and conditions. Therefore, it is related to the states and characteristics of the recipients, not the particular service providers [9].

As there is limited evidence of inequalities amongst obesity phenotypes [10–13], this study aimed to determine the socioeconomic inequality in different phenotypes of childhood obesity and its determinants in Iran.

## Methods

### Study design

The present study analyzed the combined data from the fifth national "Childhood and Adolescence Surveillance and PreventIon of Adult Noncommunicable Diseases (CASPIAN-V) Study. It was conducted in 2015 in urban and rural areas of 30 provinces of Iran. The detailed methodology has already been published [14].

### Participants

In this school-based national survey, 14,400 students, 7–18 years, were selected using multistage, stratified cluster sampling from urban and rural areas of 30 provinces of Iran in 2015. Students were sampled in each province according to their place of residence (urban or rural) and education level (elementary or high school) using probability proportional to size sampling with an equal male/female ratio. Clusters were determined at the school level. In each sampling unit, ten students alongside their parents were included. The sample size included 480 students in each province (48 clusters of 10 students).

### Data collection

The following variables were assessed:

i) Demographic information: age, sex, place of residence, family characteristics such as the family history of obesity, and parental level of education.
  Some complementary data on possessing a family private car and type of home (private/rental), some complementary information on screen time, physical activity, and other lifestyle habits were also obtained.
ii) Socio-economic status (SES):
  Family SES was calculated according to the previously approved standard method in the Progress in the International Reading Literacy (PIRLS) study [15]. Principal component analysis (PCA) determined the variables and summarized them in one principal component SES. (parents' education, parents' job, possessing a private car, school type (public/private), type of home (private/rental), and having a personal computer at home); next, SES was categorized into tertiles, in which the first tertile was the lowest SES and the third tile the highest.
iii) Screen time (ST):
  ST was considered the sum of the average daily hours spent watching TV or movies, leisure time using a personal computer (PC), or playing electronic games (EG). ST was asked separately for weekdays and weekends. ST was categorized into two groups: less than two hours per day (low) and two hours or more per day (high) according to the international ST recommendations [16].
iv) Physical activity (PA):

Two questions assessed PA: "1) during the past week, how many days were you physically active for over 30 min? (Response options: from zero to seven days); and 2) How much time do you spend in an exercise class at school per week? (Response options: from zero to three or more hours) ". A frequency of fewer than two times per week was considered as low; two to four times a week as moderate, and more than four times a week as high.

## Measurements

### Anthropometric measurement and definitions

Standardized methods were used to assess BMI [14]. Waist circumference was measured using a non-elastic tape at a point midway between the lower border of the rib cage and the iliac crest at the end of normal expiration to the nearest 0.1 cm. Generalized obesity (GO) was defined according to the WHO growth curve as BMI > 95th for age-sex specific percentile [17], and abdominal obesity (AO) was designated as waist to height ratio (WHtR) > 0.5 [18]. Students were classified into four different phenotypes of obesity in terms of AO and GO: normal (5th < BMI < 85th percentile and WHtR < 0.5), only AO (WHtR > 0.5 and BMI < 95th percentile), only GO (BMI > 95th and WHtR < 0.5), combined obesity (CO) (BMI > 95th and WHtR > 0.5).

### Statistical analysis

Data were analyzed using STATA package version 14.0 (Stata Statistical Software: Release 14. Stata Corp LP. Package, College Station, TX, USA), and a *P*-value < 0.05 was considered statistically significant.

Continuous data were presented as means (SD). The Prevalence of combinations of obesity was reported with 95% confidence intervals (CI). Univariate and multivariate logistic regression analysis assessed the association of independent variables with excess weight. The logistic regression analysis results are presented as OR (95% CI).

Although there are several methods to assess inequality in health outcomes, methods that assess inequality are not numerous. The Blinder-Oaxaca decomposition method is a well-known inequality assessment method for inequality determinants [9]. This method will divide the prevalence of the first and fifth quintile of obesity into two components. The explained or endowment component arises because of differences in the groups' characteristics, such as differences in a region or family size. An unexplained or coefficient component is attributed to different influences of these characteristics in each group [9]. In this study, using the Blinder-Oaxaca decomposition method, we assessed inequality and determinants of inequality in different phenotypes of obesity. In this method, SES inequality in different phenotypes of obesity was estimated by calculating the prevalence of different phenotypes of obesity across SES tertiles, the concentration index (C Index) [19].

C Index was estimated using the following equation, where “hi” is the value of obesity for the person “i”, “Ri” is the relative rank of person “i" in the SES variable distribution, and µ is the mean value of obesity

$$C=\frac{2}{n\upmu }\sum\limits_{i=1}^{n}hiRi-1$$

Negative and positive C Index values indicate that inequality is high and low in favor of the SES group, respectively [20].

The analysis of the obesity gap between the tertiles of SES was assessed using the Blinder-Oaxaca decomposition method [20]. in this method, two regression models fitted separately for the two population groups (in this study, upper and lower economic groups):

$$YL=\beta {X}_{L}+{\varepsilon }_{L}\mathrm{ and} YH= \beta {X}_{H}+{\varepsilon }_{H}$$

where “Y” is the outcome, “β” is the coefficient including interception, “X” is an explanatory variable, and “ε” is an error. The distance between the two groups is calculated as:s

$${\overline{Y}}_{H}-{\overline{Y}}_{L}=\left({\overline{X}}_{H}-{\overline{X}}_{L}\right){\beta }_{H}+{\overline{X}}_{L}\left({\beta }_{H}-{\beta }_{L}\right)$$

and

$${\overline{Y}}_{L}-{\overline{Y}}_{H}=\left({\overline{X}}_{H}-{\overline{X}}_{L}\right){\beta }_{L}+{\overline{X}}_{H}\left({\beta }_{H}-{\beta }_{L}\right)$$

This technique splits the gap between the mean values of a result into two components. The described component or endowment arises due to differences in the characteristics of the groups, such as differences in the area or family size. An unexplained part or coefficient is attributed to the different effects of these characteristics in each group [20].

A logistic regression model with independent variables was run for assessing decomposition in each economic group to determine the regression coefficients (β) as the main effect and their interaction with other independent variables.

This method assessed the gap decomposition in different phenotypes of obesity between SES's first and third tiles. In this study, we considered some demographic and lifestyle-related variables as determinants of different phenotypes of obesity.

Moreover, two logistic regression models (crude and adjusted) were used to assess the association of independent variables with different types of obesity. The adjusted model assessed all independent variables with the “Enter method”. To deal with the clustering effect, all analysis was performed using the”survey analysis method”.

## Results

From the 14,400 invited subjects, 14,274 students participated and completed the study (response rate: 99%). The mean (SD) age was 12.28 (3.15) years; 50.6% were boys, and 71.42% lived in urban areas.

The distribution of general characteristics of students according to gender is presented in Table 1. The frequency of high PA among boys was significantly higher than in girls (*p* < 0.001). However, the association of ST, SES, family history of obesity, and residential area with gender was not statistically significant.

**Table 1:** Demographic characteristics of students according to sex: The CASPIAN V study

	*Total*	*Boy*	*Girl*	*p-value*
*Age (year)*	*12.28 (3.15)*	*12.39 (3.14)*	*12.17 (3.16)*	< *0.001*
*Area of residence*				
* Urban*	*10,194 (71.42)*	*5150 (71.25)*	*5044 (71.59)*	*0.66*
* Rural*	*4080 (28.58)*	*2078 (28.75)*	*2002 (28.41)*	
*SES*				
* T1*	*4559 (33.46)*	*2325 (33.58)*	*2234 (33.33)*	*0.08*
* T2*	*4515 (33.14)*	*2343 (33.84)*	*2172 (32.4)*	
* T3*	*4552 (33.41)*	*2255 (32.57)*	*2297 (34.27)*	
*PA*				
* Low*	*2147 (31.92)*	*2147 (31.92)*	*2307 (35)*	< *0.001*
* Moderate*	*4424 (33.22)*	*2219 (32.99)*	*2205 (33.45)*	
* High*	*4440 (33.34)*	*2360 (35.09)*	*2080 (31.55)*	
*ST*				
* Low*	*11,644 (83.85)*	*5863 (83.44)*	*5781 (84.27)*	*0.18*
* High*	*2243 (16.15)*	*1164 (16.56)*	*1079 (15.73)*	
*FH of obesity*				
* No*	*9947 (69.69)*	*5070 (70.14)*	*4877 (69.22)*	*0.23*
* Yes*	*4327 (30.31)*	*2158 (29.86)*	*2169 (30.78)*	

*T* tertile, *SES* socioeconomic status, *PA* physical activity, *ST* screen time

**Fig. 1:**
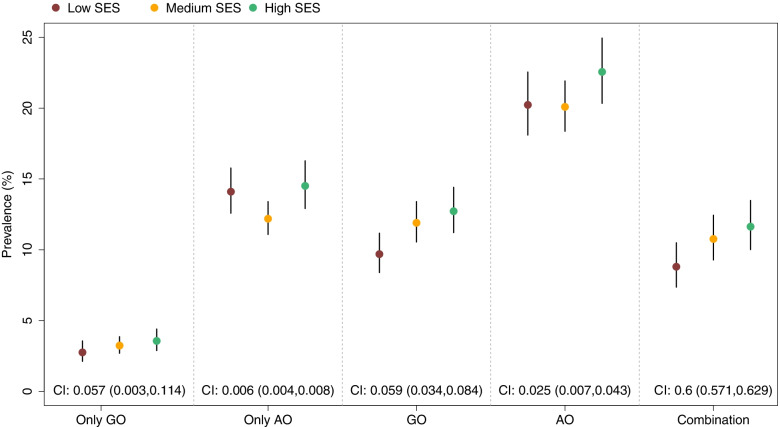
The prevalence of different phenotypes of obesity according to SES

The prevalence of AO and GO was 20.8% and 11.3%, respectively. Figure 1 shows the prevalence of different phenotypes of obesity according to SES.

Estimated values of “C Index” in this figure for different phenotypes of obesity were positive, indicating inequality was in favour of low SES groups.

Crude and adjusted association of independent variables with different phenotypes of obesity are presented in Table 2. In the adjusted model, by increasing age, the odds of "only GO" decreased significantly (OR: 0.91, 95% CI: 0.87–0.95). Moreover, having a positive family history of obesity was associated with "only GO" (OR: 1.26, 95% CI: 1.01–1.58).

**Table 2:** Association of independent variables with generalized obesity, abdominal obesity and combined obesity(GO-AO) in Iranian children and adolescents at national level in logistics regression model: The CASPIAN V study

*Variables*	*Generalized obesity only*		*Abdominal obesity only*		*Generalized obesity*		*Abdominal obesity*		*Both*	
	*Crude OR (95% CI)*	*Adjusted OR (95% CI)*	*Crude OR (95% CI)*	*Adjusted OR (95% CI)*	*Crude OR (95% CI)*	*Adjusted OR (95% CI)*	*Crude OR (95% CI)*	*Adjusted OR (95% CI)*	*Crude OR (95% CI)*	*Adjusted OR (95% CI)*
*SES (T1)*	1	1	1	1	1	1	1	1	1	1
*T2*	*1.18* *(0.93–1.50)*	*1.17* *(0.88–1.54)*	*0.85* *(0.73–0.99)*	*0.86* *(0.73–1.01)*	*1.26* *(1.07–1.49)*	*1.22* *(1.03–1.45)*	*0.99* *(0.86–1.14)*	*0.98* *(0.85–1.14)*	*1.25* *(1.01–1.54)*	*1.21* *(0.96–1.51)*
*T3*	*1.31* *(1.01–1.69)*	*1.15* *(0.88–1.52)*	*1.03* *(0.89–1.20)*	*1.01* *(0.88–1.16)*	*1.36* *(1.14–1.61)*	*1.26* *(1.07–1.49)*	*1.15* *(0.99–1.33)*	*1.10* *(0.97–1.25)*	*1.36* *(1.12–1.67)*	*1.28* *(1.06–1.55)*
*PA (low)*	1	1	1	1	1	1	1	1	1	1
*Moderate*	*1.05* *(0.84–1.32)*	*1.02* *(0.82–1.28)*	*1.04* *(0.94–1.16)*	*1.10* *(0.99–1.24)*	*1.07* *(0.93–1.23)*	*1.09* *(0.96–1.25)*	*1.06* *(0.97–1.16)*	*1.12* *(1.03–1.22)*	*1.09* *(0.93–1.27)*	*1.14* *(0.99–1.32)*
*High*	*1.12* *(0.84–1.49)*	*1.08* *(0.80–1.45)*	*0.87* *(0.76–0.99)*	*0.88* *(0.77–1.02)*	*1.01* *(0.88–1.15)*	*1.04* *(0.91–1.20)*	*0.91* *(0.82–1.01)*	*0.94* *(0.85–1.03)*	*0.96* *(0.83–1.10)*	*1.02* *(0.89–1.17)*
*Sex (girl)*	1	1	1	1	1	1	1	1	1	1
*Boy*	*1.21* *(0.86–1.69)*	*1.31* *(0.91–1.88)*	*0.98* *(0.85–1.13)*	*0.98* *(0.85–1.13)*	*1.24* *(1.06–1.46)*	*1.30* *(1.09–1.56)*	*1.07* *(0.95–1.22)*	*1.09* *(0.95–1.25)*	*1.24* *(1.03–1.48)*	*1.30* *(1.06–1.59)*
*ST (*≤ *2 h)*	1	1	1	1	1	1	1	1	1	1
> *2 h*	*1.16* *(0.87–1.54)*	*1.28* *(0.94–1.74)*	*1.07* *(0.95–1.19)*	*1.07* *(0.94–1.22)*	*1.12* *(0.96–1.31)*	*1.12* *(0.95–1.32)*	*1.08* *(0.95–1.23)*	*1.07* *(0.93–1.22)*	*1.12* *(0.93–1.35)*	*1.09* *(0.89–1.34)*
*Area of residence (urban)*	1	1	1	1	1	1	1	1	1	1
*Rural*	*0.70* *(0.49–0.99)*	*0.72* *(0.50–1.04)*	*0.79* *(0.66–0.95)*	*0.78* *(0.64–0.95)*	*0.48* *(0.40–0.58)*	*0.49* *(0.40–0.60)*	*0.62* *(0.53–0.73)*	*0.62* *(0.53–0.74)*	*0.41* *(0.34–0.50)*	*0.41* *(0.33–0.51)*
*FH of obesity (no)*	1	1	1	1	1	1	1	1	1	1
*Yes*	*1.34* *(1.10–1.65)*	*1.26* *(1.01–1.58)*	*0.95* *(0.84–1.07)*	*0.96* *(0.85–1.09)*	*1.36* *(1.16–1.59)*	*1.38* *(1.17–1.64)*	*1.09* *(0.97–1.23)*	*1.12* *(0.99–1.27)*	*1.34* *(1.10–1.64)*	*1.40* *(1.14–1.71)*
*Age (year)*	*0.91* *(0.87–0.95)*	*0.91* *(0.87–0.95)*	*1.00* *(.98–1.03)*	*0.99* *(0.97–1.02)*	*0.99* *(0.97–1.02)*	*0.98* *(0.96–1.01)*	*1.01* *(0.99–1.03)*	*1.00* *(0.98–1.02)*	*1.02* *(0.99–1.05)*	*1.01* *(0.98–1.03)*

Generalized obesity:BMI > 95^th^ age sex, Abdominal obesity: WHtR > 0.5, Only abdominal obesity: WHtR > 0.5 and 5th < BMI < 85th age sex specific percentile. Only generalized obesity: BMI > 95th age sex specific percentile and WHtR < 0.5 and Combined Obesity: BMI > 95th age sex specific percentile and WHtR > 0.5, *SES* socioeconomic status, *PA* physical activity, *FH* family history, *ST* screen time, *T* tertile

^*^ *P* ≤ 0.05 is considered as significant

In the multivariate model, living in rural areas decreased the odds of "only AO" (OR: 0.78, 95% CI: 0.64–0.95).

In the adjusted model, students with high SES (T2: OR: 1.22 95% CI:1.03–1.45, T3:OR: 1.26 95% CI: 1.07–1.49), being male (OR: 1.30 95% CI:1.09–1.56), living in rural areas (OR: 0.49 95% CI:0.40–0.60), and positive family history of obesity (OR: 1.38 95% CI:1.17–1.64) was associated with "GO". Moderate physical activity (OR: 1.12 95% CI: 1.03–1.22) increased, and living in a rural area (OR: 0.62 95% CI: 0.53–0.74) decreased the odds of "AO". In addition, living in rural areas (OR: 0.41, 95% CI: 0.33–0.51), high SES (OR: 1.28, 95% CI: 1.06–1.55), being male (OR: 1.30, 95% CI: 1.06–1.59) and positive family history of obesity (OR: 1.40, 95% CI: 1.14–1.71) was associated with combined obesity in the adjusted model.

Table 3 shows that the gap between the low and high SES groups for the prevalence of combination obesity (GO –AO) and GO was 3.26%, and 3.27%, respectively, but generalized (β: -0.63, CI:-1.47, 0.21) and abdominal obesity (β: 0.57, CI:-2.17, 1.03) were not significant. In the explained component, place of residence significantly contributed to the gap between the two SES groups for the prevalence of combined obesity. For general obesity, place of residence and family history of obesity were the effective variables responsible for the gap.

**Table 3:** Decomposition of the gap in generalized obesity, abdominal obesity and combined obesity(GO-AO) prevalence between the first and third tiles of socioeconomic status in Iranian children and adolescents: The CASPIAN V study

	*Generalized obesity only*	*Abdominal obesity only*	*Generalized obesity*	*Abdominal obesity*	*Both*
Prevalence, *% (95% CI)*					
* Prevalence in first tile*	*2.77 (2.21,3.33)*	*13.96 (12.85,15.08)*	*9.49 (8.6,10.39)*	*19.93 (18.71–21.15)*	*8.55 (7.62,9.48)*
* Prevalence in third tile*	*3.40 (2.77,4.03)*	*14.53 (13.38,15.68)*	*12.77 (11.75,13.79)*	*22.73 (21.45,24.01)*	*11.81 (10.73,12.88)*
* Differences (total gap)*	*-0.63 (-1.47,0.21)*	*-0.57 (-2.17,1.03)*	*-3.27 (-4.63,-1.92)*	*-2.80 (-4.57,-1.03)*	*-3.26 (-4.67,-1.84)*
*Due to endowments (explained), β (95% CI)*					
* PA*	*0.02 (-0.03,0.07)*	*-0.05 (-0.15,0.06)*	*0.06 (-0.01,0.14)*	*0 (-0.11,0.11)*	*0.06 (-0.02,0.14)*
* Sex*	*0.03 (-0.01,0.06)*	*0 (-0.03,0.03)*	*0.04 (-0.03,0.11)*	*0.02 (-0.02,0.07)*	*0.03 (-0.03,0.1)*
* ST*	*-0.02 (-0.06,0.01)*	*-0.01 (-0.08,0.05)*	*-0.06 (-0.12,0.01)*	*-0.04 (-0.11,0.03)*	*-0.05 (-0.11,0.01)*
* Area of residence*	*-0.11 (-0.25,0.04)*	*-0.51 (-0.79,-0.23)*	*-0.94 (-1.18,-0.7)*	*-1.21 (-1.53,-0.88)*	*-1.05 (-1.31,-0.8)*
* FH of obesity*	*-0.02 (-0.05,0.01)*	*0.01 (-0.05,0.07)*	*-0.12 (-0.2,-0.03)*	*-0.07 (-0.14,0.01)*	*-0.11 (-0.2,-0.02)*
* Age*	*-0.03 (-0.07,0.02)*	*-0.01 (-0.04,0.02)*	*-0.01 (-0.03,0.01)*	*0.01 (-0.02,0.04)*	*0.02 (-0.02,0.06)*
* Subtotal*	*-0.13 (-0.3,0.03)*	*-0.57 (-0.88,-0.26)*	*-1.01 (-1.29,-0.73)*	*-1.28 (-1.64,-0.93)*	*-1.1 (-1.4,-0.81)*
*Due to coefficients (unexplained), β (95% CI)*					
* PA*	*-0.16 (-1.4,1.08)*	*-0.95 (-3.23,1.34)*	*-0.52 (-2.51,1.47)*	*-1.03 (-3.57,1.52)*	*-0.44 (-2.47,1.58)*
* Sex*	*0.7 (-0.14,1.54)*	*-0.35 (-1.93,1.23)*	*-0.58 (-1.93,0.78)*	*-1.38 (-3.15,0.39)*	*-1.35 (-2.76,0.06)*
* ST*	*0.23 (-0.16,0.62)*	*-0.17 (-0.87,0.52)*	*0.41 (-0.21,1.04)*	*0.02 (-0.76,0.8)*	*0.25 (-0.39,0.9)*
* Area of residence*	*1.22 (-1.16,3.6)*	*1.35 (-3.12,5.82)*	*-1.41 (-4.97,2.14)*	*-1.13 (-5.97,3.72)*	*-2.61 (-6.18,0.97)*
* FH of obesity*	*0.33 (-0.27,0.92)*	*-0.24 (-1.32,0.84)*	*-0.16 (-1.13,0.8)*	*-0.57 (-1.8,0.66)*	*-0.41 (-1.42,0.61)*
* Age*	*-2.47 (-5.86,0.91)*	*-1.36 (-7.75,5.02)*	*-3.51 (-8.71,1.69)*	*-2.62 (-9.57,4.32)*	*-2.51 (-7.91,2.89)*
* Subtotal*	*-0.35 (-5.19,4.5)*	*1.72 (-7.28,10.73)*	*3.51 (-3.9,10.92)*	*5.2 (-4.65,15.05)*	*4.91 (-2.7,12.52)*

Only abdominal obesity: WHtR > 0.5 and 5th < BMI < 85th age sex specific percentile, Only generalized obesity: BMI > 95th age sex specific percentile and WHtR < 0.5 and Combined Obesity: BMI > 95th age sex specific percentile and WHtR > 0.5, *SES* socioeconomic statu, *PA* physical activity, *FH* family history, *ST* screen time

^*^ *P* ≤ 0.05 is considered as significant

## Discussion

This study shows that the prevalence of obesity is higher in groups with high SES. This finding is consistent with previous studies [10, 3, 21].

Socioeconomic inequality in the health of children and adolescents is one of the most critical health concerns in any country [22]. Studies show that developed countries witness a weakening of the positive relationship between obesity and SES, and a gradual increase in their negative relationship [23]. However, developing countries like Iran report that the prevalence of childhood obesity is associated with high levels of socioeconomic inequality [21].

The epidemiological transition in low and middle-income countries (LMICs), where energy-dense foods are readily available at relatively low prices, is high [24, 25]. In Iran, increasing access to high-calorie foods and a sedentary lifestyle are the two major causes of obesity [21].

Georgina Gómez et al. found that SES matches the quality and variety of food [26]. A multilevel study on childhood determinants of obesity (2019) reported family income and the amount of child allowance was related to obesity and weight gain [27].

In many Asian cultures, obesity in children is a sign of their health in affluent families [28], and this belief can explain the prevalence of obesity in families with favourable socioeconomic status. SES has been raised as a fundamental determinant of adult health, although confirmation of this relationship in children requires further studies [29].

Our findings show that the prevalence of AO and GO were 20.8% and 11.3%, respectively, consistent with previous Iranian studies. For example, in a survey in northwestern Iran, the prevalence of GO and AO was 26.6% and 43.4%, respectively [30]. In Azerbaijan, the prevalence of GO and AO was 24% and 76.4%, respectively [31]. These results confirmed that AO in Iranian children and adolescents is more prevalent than GO.

According to the present findings, gender is a risk factor for combined obesity (AO, GO) and GO, such that the risk of this phenotype of obesity in boys is higher than in girls. Moreover, previous studies expressed the relationship between GO and AO with gender [21, 32]. Similarly, in a study of obesity trends in Chinese children between 2011 and 2015, obesity was higher among boys compared to girls [33]. However, in some studies, the results were different, and obesity was higher among girls compared to boys [34].

These differences can be attributed to cultural differences that attended to boys more than girls. For example, the prevalence of obesity and overweight in Iranian families is more common due to increased attention to boys [35]. Other causes of gender differences in obesity phenotypes include differences in lifestyle, socio-individual characteristics, and genetic and behavioural characteristics. However, in the present study, no significant relationship was observed between “only AO” and “only GO” with gender; no other studies have investigated their prevalence. This finding expresses the need to address obesity phenotypes as a cumulative indicator to expand the obesity pattern by adding and combining its phenotypes [36].

According to the results of the Oaxaca model, the highest proportion of differences between rich and poor groups regarded the living area, pertaining that urbanization was associated with a high prevalence of obesity phenotypes. Environmental factors such as place of residence and socioeconomic level, influenced children and adolescents' food consumption patterns and eating habits.

Differences in urban and rural food cultures are leading causes of these inequalities. Using traditional foods and living a more active life than ready-made and sedentary foods increases inequalities in obesity phenotypes [37].

The human–environment relationship is complex and requires a comprehensive method to create transformational change in health; because the environment directly or indirectly affects occupant behaviour; evidence suggests that with the proper lifestyle changes of improved nutritional intake and increased physical activity, obesity is a preventable disease [38]; thus indicating the importance of preventive measures.

### Strengths and limitations

The present study is the first to compare different phenotypes of childhood obesity based on socioeconomic levels by the Blinder-Oaxaca decomposition model. This model shows the proportion of each determinant in creating inequality [29].

Furthermore, our large sample size, using standard protocols and a validated questionnaire, is another strength of the present study; However, since it is cross-sectional, it can not express the causal relationship between the variables, which is one of the limitations of the recent research. In addition, in this study, we didn't assess Fat mass, which is another limitation. Therefore, prospective studies in this field are recommended.

## Conclusion

According to the present study findings, all childhood obesity phenotypes were more prevalent in Iranian children with high SES. Therefore, due to obesity and other diseases, it is essential to implement environmental changes in addition to designing macro-educational programs and prevention strategies.

## Data Availability

The data that support the findings of this study are available from the corresponding author upon reasonable request.
